# Clinical and Ultrasonographic Characteristics of the Achilles Tendon in Hemodialysis Patients

**DOI:** 10.3390/medicina59122181

**Published:** 2023-12-15

**Authors:** Samar Tharwat, Marwa Saleh, Rabab Elrefaey, Mona Kamal Nassar, Mohammed Kamal Nassar

**Affiliations:** 1 Rheumatology & Immunology Unit, Department of Internal Medicine, Faculty of Medicine, Mansoura University, Mansoura 35516, Egypt; 2 Department of Internal Medicine, Faculty of Medicine, Horus University, New Damietta 34517, Egypt; m_kamal@mans.edu.eg; 3 Mansoura Nephrology & Dialysis Unit (MNDU), Department of Internal Medicine, Faculty of Medicine, Mansoura University, Mansoura 35516, Egypt; marwasaleh@mans.edu.eg (M.S.); rababelrefaey@yahoo.com (R.E.); 4 Department of Radiology, Student Hospital, Mansoura University, Mansoura 35516, Egypt; manmooooonnassar@yahoo.com

**Keywords:** Achilles tendon, tendinosis, enthesopathy, ultrasound, hemodialysis

## Abstract

*Background and Objectives*: The early recognition of tendon alterations in chronic hemodialysis (HD) patients, an awareness of the factors that influence the condition, and active intervention have considerable clinical relevance. The aim of this study was to investigate the musculoskeletal ultrasound (MSUS) features of the Achilles tendon in chronic HD patients and determine the factors associated with tendon abnormalities. *Materials and Methods*: This study was conducted on 46 HD patients and 24 sex- and age-matched controls. All participants were evaluated clinically for any signs of Achilles tendon abnormalities. Then, the Achilles tendon was scanned bilaterally using MSUS. *Results*: Among the 92 Achilles tendons in the HD patients, there was tenderness and swelling of only two (2.2%). Regarding MSUS features, there were statistically significant higher thicknesses in the proximal end (*p* < 0.001), midpoint (*p* < 0.001), and distal end (*p* < 0.001) of the Achilles tendons in the HD patients when compared with the healthy controls. Tendinosis was found in 12 (13%) of the HD patients’ Achilles tendons, which was statistically significant in comparison to the healthy controls (*p* = 0.008). There were statistically significant higher scores of structural abnormalities (*p* = 0.005), bone erosions (*p* = 0.017), and calcifications (*p* = 0.015) in the HD patients when compared to the healthy controls. According to the results of a univariate regression analysis, age and male gender were predictive for US abnormalities in HD patients (*p* = 0.002 and 0.025, respectively). *Conclusions*: The Achilles tendon in subjects on chronic HD showed frequent US abnormalities. These abnormalities in HD patients appear to be more related to age and gender and may be asymptomatic.

## 1. Introduction

Hemodialysis (HD) is an essential and widely utilized renal replacement therapy (RRT) for patients with end-stage renal disease (ESRD) [1]. According to the International Society of Nephrology, 2.62 million individuals worldwide received RRT to treat ESRD; the majority were treated with HD [2]. Even as the treatment with HD advances, complications, such as renal hypertension and musculoskeletal manifestations, continue to occur and have a negative impact on the patients’ health-related quality of life (HRQoL) [3].

Maintaining the integrity of the tendons is critical for achieving optimal force transfer from the muscles and functional capacity [4]. Spontaneous tendon rupture is a rare complication that may occur during physical exercise; chronic metabolic disorders, such as chronic renal failure (CRF), diabetes mellitus, systemic lupus erythematosus, rheumatoid arthritis, gout, and obesity, have all been linked to it [5].

ESRD patients experience a gradual loss in muscle mass, strength, and overall physical capability [6]. The precise mechanism that is responsible for tendon abnormalities in HD patients is still debated, and it has evolved from connective tissue elastosis to a multifactorial compound that is predominantly influenced by secondary hyperparathyroidism as a result of long-term HD treatment [7]. Additionally, patients who have CRF are more likely to have chronic acidosis, an accumulation of uremic toxin, malnutrition, b2-amyloidosis, and poor collagen metabolism, all of which have the potential to contribute to the development of spontaneous tendon rupture [8]. The Achilles tendon is the thickest and strongest tendon in the body, yet it is prone to failure and rupture in HD, which is thought to be due to changes in the elastic characteristics of the tendon. Patients on long-term HD commonly encounter disruptions in calcium–phosphorus metabolism, leading to the development of secondary hyperparathyroidism. An excess of parathormone causes degradation of tendon tissue, whereas a decrease in active vitamin D receptors in the parathyroid gland leads to a low level of active vitamin D, resulting in the denaturation of ligament and tendon tissue. Furthermore, metabolic acidosis is also responsible for the disruption of collagen synthesis [9]. In general, these circumstances result in atypical collagen constituents in tendon tissues.

Over the years, the applications for musculoskeletal ultrasound (MSUS) have expanded substantially, making MSUS an effective, less-expensive alternative to MRI in many clinical settings [10]. MSUS has no contraindications, such as cardiac pacemakers and other body implants, in contrast to MRI. Moreover, MSUS has a higher spatial resolution than MRI, allowing for the detection of finer anatomical details. It is also unique in its ability to perform dynamic imaging, correlate findings with the patient’s symptomatology, and guide procedures that enable diagnosis and treatment to be accomplished in a single clinical session [11].

In medical practice, grayscale US (GSUS) is frequently employed in the examination of a suspected tendon pathology [12]. In many tendons, such as the Achilles tendon, GSUS has been shown to have diagnostic accuracy and sensitivity greater than 90% for each [13].

As HD improves, the long-term survival of patients with ESRD and the quality of life of ESRD patients on chronic HD have consequently received more attention [3]. Some patients with ESRD suffer from tendon degeneration, while tendon rupture may appear as the disease progresses, severely impacting their daily activities and quality of life [14]. When it comes to the prevention of severe tendinopathy, the early recognition of tendon alterations in HD patients, an awareness of the factors that influence the condition, and active intervention all have considerable clinical relevance.

The aim of this study was to investigate the GSUS features of the Achilles tendon in ESRD patients who were receiving chronic HD and determine the factors associated with tendon abnormalities.

## 2. Materials and Methods

### 2.1. Study Population

This cross-sectional observational study was conducted at Mansoura Nephrology and Dialysis Unit (MNDU), Mansoura University Hospital, Egypt, between January and August 2023 on 46 patients with ESRD receiving HD. The sample size was selected as a convenience sample. We used convenience sampling because it is usually low-cost and easy with subjects readily available [14]. All patients who met the inclusion criteria were offered the opportunity to participate in the study unless they met any of the exclusion criteria or declined. Patients diagnosed with ESRD who were at least 18 years of age at the time of the study and had been receiving HD treatment for more than 6 months at a frequency of three times per week were considered eligible for participation. Patients were excluded from the study if they had a history of seronegative arthropathy, active infection, psoriasis, peripheral neuropathy, chronic use of steroids and/or fluoroquinolone medication during the past 6 months, severe trauma, or ankle joint surgery. Twenty-four healthy controls matched for age and gender were also included. This study was carried out in conformity with the principles outlined in the Declaration of Helsinki. It was approved by the Institutional Research Board of Mansoura University’s Faculty of Medicine (Approval number: R.23.06.2227). Comprehensive information regarding this study was provided to all participants. Informed consent was obtained either directly from the participants themselves or from the legal guardians of patients lacking formal education.

### 2.2. Data Collection

We gathered sociodemographic information, such as age, gender, occupation, lifestyle, socioeconomic status, and smoking habit. The height and weight of each person were taken into account to figure out the body mass index. The electronic and written medical records of each HD patient were searched for therapeutic data and clinical characteristics, such as the cause for HD and the length of time the patient had been receiving HD.

### 2.3. Clinical Evaluation of the Achilles Tendon 

An experienced rheumatologist carried out clinical examinations on all HD patients as well as healthy controls to identify any clinical manifestations that might point to Achilles tendon pathology. These manifestations included pain, swelling, or tenderness that was brought on by mobilization, pressure, or contraction against resistance. The clinician identified the intratendinous swelling in the tendon using the Arc sign by having the patient actively dorsiflex and plantarflex the ankle joint while observing the swelling movement between the malleoli [15]. The clinician assessed tenderness using the Royal London test [16] by palpating the tendon with the ankle in either a neutral position or slight plantar flexion in order to identify any local tenderness. Following this, the ankle was dorsiflexed and plantarflexed actively. A confirmation palpation was performed on the tender portion of the tendon while the ankle was in a state of maximal dorsiflexion.

### 2.4. Musculoskeletal Ultrasound of the Achilles Tendon 

All US examinations were performed by a rheumatologist who possessed a minimum of eight years of experience working in the field of musculoskeletal ultrasonography (MSUS). At the time of the US examination, the rheumatologist was blind to the clinical evaluation of the Achilles tendon. In this study, EDAN U2 ultrasound equipment from Shenzhen, China, equipped with a linear array transducer, was used. The frequency range was 8 to 13.4 MHz. The frequency was set to 13 MHz, and the sonographic settings were tweaked so that the resulting scans would yield the clearest possible images of the Achilles tendon.

Between the skin and the probe, a stand-off gel pad was placed. The subjects were lying prone on the examining table with both feet permitted to swing down freely from the end of the table. The ankle joint was held at a 90-degree angle. The Achilles tendon was scanned with ultrasound on both sides from its proximal end to its insertion at the back of the calcaneus. At the axial view, the thickness of the Achilles was measured as the maximal anteroposterior diameter at three points: the proximal end, the distal end, and the midpoint in between. Tendinosis was characterized by the presence of a hypoechoic aspect of the tendon, a loss of fibrillar pattern, or a fusiform thickness.

In addition, the Achilles entheses were examined to determine whether or not there were any structural abnormalities, retrocalcaneal bursitis, bone erosions, or calcification.

### 2.5. Blood Sampling and Laboratory Tests 

Blood samples were drawn from the HD patients’ vascular access just moments before the beginning of the first HD session of the week. On the days of blood sampling, laboratory analyses were carried out using an automated analyzer. The following laboratory tests were performed: complete blood count (CBC), serum calcium (Ca), phosphorus (PO4), intact parathyroid hormone level (iPTH), ferritin, and transferrin saturation.

### 2.6. Statistical Analysis 

On a personal computer, data were gathered and analyzed with IBM SPSS Statistics version 24.0 for Windows (IBM Corp., Armonk, NY, USA). Numbers of cases and percentages (%) were used to show qualitative variables, whereas medians (minimum–maximum) or means ± standard deviation were employed for all quantitative data. The significance of differences between the two groups was evaluated using the independent samples t-test for normally distributed variables or the Mann–Whitney test for non-parametric variables. The Shapiro–Wilk test was used to check for normality in the distribution of continuous variables. The chi-square test or Fisher exact test was used as necessary for comparisons between qualitative variables. In order to identify important variables that were associated with Achilles tendon abnormalities, univariate correlation analysis utilizing the Spearman’s test was conducted. Univariate linear regression analysis was then conducted to determine the predictors of abnormalities in the Achilles tendon. A *p* value under 0.05 was regarded as significant.

## 3. Results

**Figure 1:**
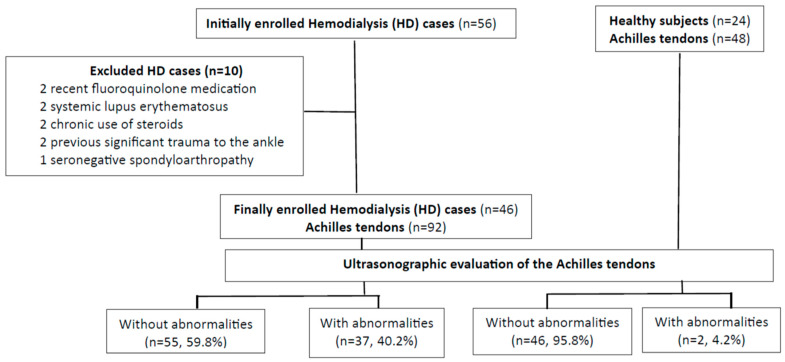
Flowchart of this study.

This study included a total of 46 HD patients with a mean age of 52.34 ± 14.99 years (25 males and 21 females) and 24 sex- and age-matched healthy controls with a mean age of 51.21 ± 13.22 years (13 males and 11 females). We scanned 92 Achilles tendons in the HD patients and 48 in the healthy controls. The flowchart of this study is illustrated in Figure 1.

The sociodemographic data, clinical features, and laboratory findings associated with the HD patients are presented in Table 1. The median duration of HD was three years, and the most common associated comorbidity was hypertension (54.3%). The median value for serum calcium was 8.5 mg/dL, phosphorus was 5.4 mg/dL, intact PTH was 531 pg/mL, hemoglobin level was 11.2 g/dL, serum ferritin was 263.4 ng/dL, and transferrin saturation was 21%.

As shown in Table 2, clinical examination revealed tenderness and swelling of only 2 (2.2%) of 92 Achilles tendons in the HD patients. Regarding US features, there were statistically significant higher thicknesses in the proximal end (*p* < 0.001), midpoint (*p* < 0.001), and distal end (*p* < 0.001) of the Achilles tendons in the HD patients when compared with the healthy controls. Additionally, tendinosis was found in 12 (13%) of the HD patients’ Achilles tendons, which was statistically significant in comparison to the healthy controls (*p* = 0.008). Regarding Achilles tendon entheses, there were statistically significant higher scores of structural abnormalities (*p* = 0.005), bone erosions (*p* = 0.017), and calcifications (*p* = 0.015) in the HD patients when compared to the healthy controls.

**Figure 2:**
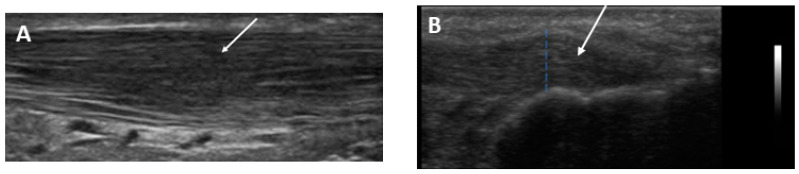
Longitudinal US scan of the Achilles tendon showing heterogenous echogenicity of the tendon with loss of fibrillar pattern and fusiform thickness suggestive of tendinosis (**A**) at the midpoint (arrow) in a 52-year-old female patient with 10 years hemodialysis duration and (**B**) at the insertion site of the tendon (dotted blue line and arrow) in a 60-year-old male patient with 13 years hemodialysis duration.

**Figure 3:**
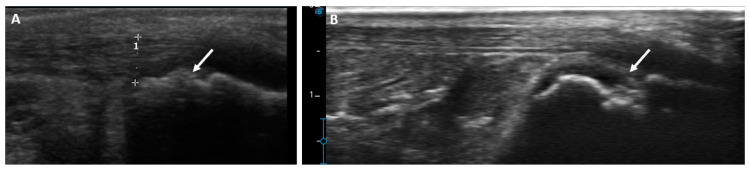
Longitudinal US scan of the Achilles tendon showing erosion (arrows) on insertion (**A**) in a 30-year-old male with 6 years hemodialysis duration and (**B**) in a 57-year-old male patient with 8 years hemodialysis duration.

**Figure 4:**
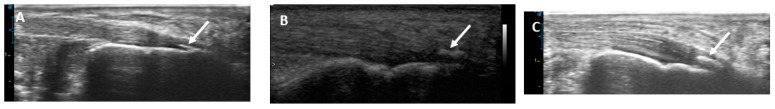
Longitudinal US scan of the Achilles tendon showing calcifications at the site of distal insertion of the tendon (arrows); (**A**) mild in a 30-year-old female patient with 2 years hemodialysis duration, (**B**) moderate in a 54-year-old male patient with 8 years hemodialysis duration, (**C**) moderate in a 70-year-old male patient with 5 years hemodialysis duration.

**Figure 5:**
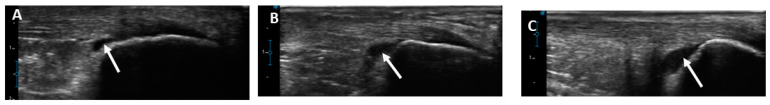
Longitudinal US scan of the Achilles tendon showing retrocalcaneal bursitis of different sizes (arrows); (**A**) small in a 35-year-old male patient with 2 years hemodialysis duration, (**B**) medium in a 34-year-old female patient with 1 year hemodialysis duration, (**C**) medium in a 62-year-old male patient with 10 years hemodialysis duration.

Demonstrative scans of the Achilles tendon US abnormalities in this study’s HD patients are presented in Figure 2, Figure 3, Figure 4 and Figure 5.

**Figure 6:**
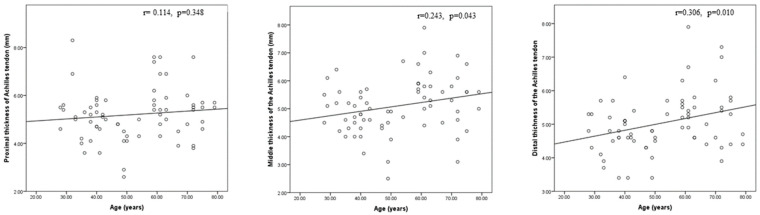
Correlation between age and proximal, middle, and distal thicknesses of the Achilles tendon in this study’s hemodialysis patients.

**Figure 7:**
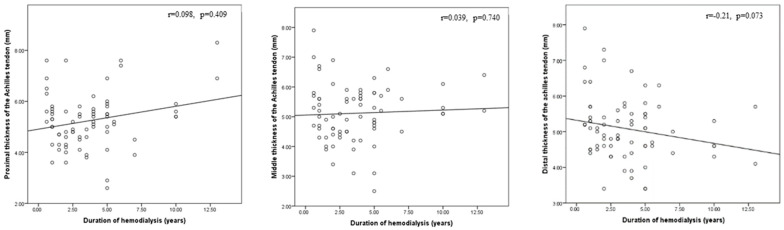
Correlation between duration of hemodialysis and proximal, middle, and distal thicknesses of the Achilles tendon in this study’s hemodialysis patients.

**Figure 8:**
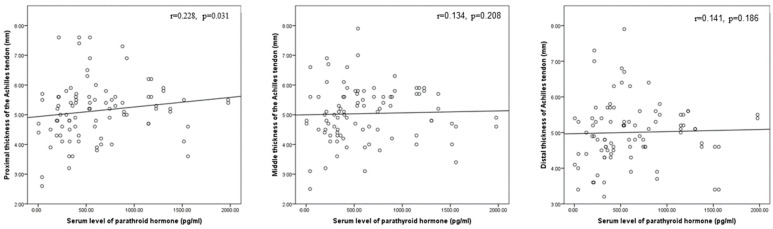
Correlation between serum parathyroid hormone and proximal, middle, and distal thicknesses of the Achilles tendon in this study’s hemodialysis patients.

As illustrated in Figure 6, Figure 7 and Figure 8, there was a statistically significant positive correlation between age and thickness and both midpoint thickness (*r* = 0.243, *p* = 0.043) and distal-end thickness (*r* = 0.306, *p* = 0.010) of the Achilles tendon in the HD patents. Additionally, there was a statistically significant positive correlation between iPTH and proximal-end thickness of the Achilles tendon in the HD patients (*r* = 0.228, *p* = 0.031).

According to the results of a univariate regression analysis, age and male gender were predictive for US abnormalities in the HD patients (*p* = 0.002 and 0.025, respectively), although weight, height, iPTH, and serum calcium level had shown no statistically significant association, as shown in Table 3.

## 4. Discussion

The aim of this study was to identify Achilles tendon abnormalities in chronic HD patients based on clinical manifestations and MSUS findings. We performed rheumatological evaluations and MSUS scans on 92 Achilles tendons in chronic HD patients and compared them to 48 Achilles tendons in age- and sex-matched controls. Our results demonstrated that MSUS findings indicative of Achilles tendon abnormalities and enthesopathy were more common in the chronic HD patients compared to the age- and sex-matched healthy controls. These abnormalities may be subtle and subclinical. Age and male gender were predictive of these abnormalities.

In this study, we found that the prevalence of MSUS abnormalities was correlated with much higher clinical manifestations. In fact, subclinical musculoskeletal conditions, such as asymptomatic subclinical enthesopathy, have been identified in HD patients [17].

In the current study, there was a statistically significant higher thickness of the Achilles tendons in the HD patients when compared with the healthy controls. Tendon thickening is caused by the deposition of amyloid tissue on the tendon’s deep and superficial surfaces [18].

Tendinosis, which can result in a tendon rupturing on its own, is linked to long-term HD as well as kidney transplantation [19,20,21]. In the current study, Achilles tendon tendinosis was demonstrated in 13% of the scanned tendons in the HD patients, which was significantly higher than the healthy controls. Tendon abnormalities, such as calcific deposition, increased thickness, and abnormal peritendon tissue, were also identified in another study that assessed the quadriceps and Achilles tendons of 50 HD patients using MSUS [22]. In this regard, Rahatli FK and coworkers measured the thickness and shear wave velocity (SWV) of the middle thirds of the Achilles tendons in a study with twenty-five patients who were on chronic HD for at least five years, twenty-five renal transplant patients, and twenty-five healthy controls. The patients with CRF had Achilles tendons that were softer than the patients with renal transplants and the control group. This was related to chronic tendinopathy, which causes tendon softening and weakening [23]. 

The region at which ligaments, tendons, or joint capsules are attached to bone is referred to as the enthesis. Due to its often-subclinical presentation, the prevalence of enthesitis among patients undergoing HD is likely underestimated [24]. In HD patients, it has been shown that the Achilles tendon is one of the entheses that become implicated most frequently. In the present study, there were statistically significant higher scores of structural abnormalities, bone erosions, and calcifications at the scanned entheses of the Achilles tendon in the HD patients when compared to the healthy controls. This is consistent with the Brountzos et al. study [25] in which they evaluated the Achilles tendon, although they only used grayscale assessment and a low-frequency probe (7–7.5 MHz). The findings of their study showed a significantly higher prevalence of enthesopathy in dialysis patients compared to healthy controls.

There are several different hypotheses about the pathogenesis of tendinopathy in general. Some of these theories include the vascular and neurogenic theory [26], the apoptosis theory [27], the mechanical theory [28], the inflammatory theory [29], and the continuum model [30]. For the purpose of guiding treatment decisions for the clinical presentations of tendinopathy, the continuum model of tendon disease was conceived with the intention of taking into account both clinical symptoms and laboratory-based research. The model has three distinct stages, namely reactive tendinopathy, tendon disrepair (characterized by unsuccessful tendon healing), and degenerative tendinopathy. Several previous research studies attempted to integrate these various elements into a three-stage framework comprising injury, unsuccessful healing, and the manifestation of clinical symptoms [31].

According to the results of a univariate regression analysis in our cohort, age and male gender were predictive for MSUS abnormalities in the HD patients. In a study that was conducted on 45 elderly (age 65 years) and 42 young (age 18–40 years) healthy controls to investigate age-related alterations in Achilles tendons using sonoelastography, the results revealed increased tendon stiffness in the elderly subjects, which may be responsible for the high prevalence of Achilles tendinopathies in elderly subjects [32]. Through the process of ageing, certain molecular changes take place in the structure and composition of tendons, just as they happen in the other tissues, and these changes have the potential to make the tendon stiffer [33,34]. Achilles tendinopathy is a prevalent clinical condition that primarily affects elderly individuals. It is believed that overuse and repetitive loading contribute to tendinopathy. In the elderly, decreased ankle dorsiflexion range of motion, abnormal subtalar range of motion, decreased plantar flexion strength, excessive foot pronation, and inadequate tendon vascularization are believed to be intrinsic risk factors that affect the Achilles tendon [35]. On the other hand, the findings of a number of clinical studies showed that there was no significant link between age and the degree of stiffness of the Achilles tendon in healthy adults of varying ages [36,37].

Patellar and Achilles tendon ruptures can happen in persons who have secondary or tertiary hyperparathyroidism. Tertiary hyperparathyroidism is distinguished from secondary hyperparathyroidism in that it is characterized by an autonomous parathyroid function that is independent of serum calcium concentration. In this instance, the parathormone (PTH) levels are extremely elevated, and the clinical pattern is characterized by widespread osteoporosis with sub-periosteal bone resorption and the resultant weakening of the bone–tendon junction [38].

Weerakkody and Gaillard reported that hyperparathyroidism is one of the causes of Achilles tendon tears, manifested by pain and swelling in the Achilles region. Ultrasound reveals an enlargement of the tendon thickness with abnormally hypoechoic regions, which is most associated with tendinosis, or may reveal separation of the tear ends with a contour change in the tendon in the case of a full-thickness tear [39]. Terai et al. found that CKD patients with secondary hyperparathyroidism and hyperphosphatemia had more calcification in their blood vessels. This may be the same thing that causes calcium to deposit in tendons [40]. In a study of 50 ESRD patients on chronic HD who underwent quadricep tendon and Achilles tendon US, PTH was found to be positively linked with the presence of a calcific deposit and increased Achilles tendon thickness [22]. In our study, there was a statistically significant positive correlation between iPTH and proximal-end thickness of the Achilles tendon in the HD patients. This may explain tendon rupture in HD patients with hyperparathyroidism.

Male gender was also found to be a risk factor for Achilles tendon abnormalities in our cohort. Males were found to have stiffer patellar tendon, medial gastrocnemius, rectus femoris, and gastrocnemius muscles [41,42,43,44]. However, other studies indicated that the Achilles tendon [45,46], patellar tendon [47], medial gastrocnemius muscle [48], and biceps brachia muscle [43] had similar stiffness in males and females. Others revealed that tricep surae [49] and bicep brachia [50] muscular stiffness was higher in females than that in males.

It is estimated that more than one-third of patients with CRF have Achilles tendon abnormalities after a mean duration of HD of six years [25]. In a previous study conducted on 49 HD patients using the Glasgow Ultrasound Enthesitis Scoring System (GUESS) to evaluate the entheseal sites of the lower extremities in HD patients and to correlate the findings with the duration of HD, the thickness of the quadricep and Achilles tendons and plantar fascia increased with the duration of HD [51]. However, we found no statistically significant association between Achilles tendon abnormalities and HD duration.

This study had several limitations. A major limitation was the small sample size in both groups. Also, the design of our study, which was cross-sectional, is an important limitation of this work. It would have been better if we used elastography to evaluate the elastic characteristics of the scanned Achilles tendons. Additionally, this study was based on convenience sampling, which is a non-probability sampling method that involves selecting participants based on their availability and accessibility that may be associated with sampling bias and lack of randomness. Lastly, the number of participants in the control group was less than that of the case group, which should be considered in further studies. Longitudinal studies are required to determine if these abnormalities progress to symptomatic tendinopathy or calcaneal spur development.

## 5. Conclusions

In conclusion, the Achilles tendon in the subjects on chronic HD showed frequent US abnormalities. These abnormalities may be subtle and subclinical. The Achilles tendon abnormalities in HD patients appear to be more related to age and gender. Ultrasound scanning has the potential to serve as an easily accessible, cost-effective, and safe tool for evaluating suspected Achilles tendon pathology in HD patients. 

## Figures and Tables

**Table 1:** Sociodemographic data and clinical and laboratory characteristics of this study’s HD patients (n = 46).

Variable n (%), Mean ± SD, Median (Min–Max)	HD Patients (n = 46)
Sociodemographic data	
Age, years	52.34 ± 14.99
Gender	
Male	25 (54.3)
Female	21 (45.7)
Occupation	
Not employed	24 (52.2)
Employed	9 (19.6)
Retired	6 (13)
Not able to work due to disability	7 (15.2)
Active lifestyle	10 (21.7)
Socioeconomic level	
Low	21 (45.7)
Average	21 (45.7)
High	4 (8.7)
Smoking	
Never	33 (71.7)
Former smoker	12 (26.1)
Current Smoker	1 (2.2)
Anthropometric measures	
Weight, kg	84.34 ± 17.72
Height, m	168.11 ± 6.94
Body mass index, kg/m^2^	29.93 ± 6.42
Clinical data	
Cause of chronic renal failure	
ADPKD	1 (2.2)
Drug induced	2 (4.3)
Glomerulonephritis	3 (6.5)
Hypertension	8 (17.4)
Diabetes Miletus	3 (6.5)
Neurogenic bladder	1 (2.2)
Obstructive uropathy	1 (2.2)
Unknown cause	14 (30.4)
Duration of HD, years	3 (0.6–13)
Associated comorbidities	
Diabetes Miletus	5 (10.9)
Hypertension	25 (54.3)
Ischemic heart disease	4 (8.6)
Therapeutic data	
Erythropoietin	32 (69.6)
Calcium supplementation	27 (58.7)
Iron supplementation	22 (47.8)
Antihypertensives	20 (43.5)
Antidiabetics	5 (10.9)
Laboratory data	
Calcium, mg/dL	8.5 (5.20–9.7)
Phosphorus, mg/dL	5.4 (1.9–17.5)
Intact PTH, pg/mL	531 (4.6–1978)
Hemoglobin, g/dL	11.2 (8–14.3)
Serum ferritin, ng/mL	263.4 (12.6–1730)
Transferrin saturation, %	21 (5–43)

**Table 2:** Clinical findings and ultrasonographic features of the Achilles tendon in this study’s HD patients (n = 48) and healthy subjects (n = 92).

Variable n (%), Mean ±SD, Median (Min–Max)	Healthy Subjects (n = 24) (Achilles Tendons = 48)	Hemodialysis Patients (n = 46) (Achilles Tendons = 92)	*p*
Clinical findings			
Pain	4 (8.3)	3 (3.3)	0.231
Swelling	0	2 (2.2)	0.546
Tenderness	0	2 (2.2)	0.546
Ultrasonographic features			
Thickness, mm			
Proximal	3.84 ± 0.86	5.19 ± 1.05	<0.001 *
Middle	3.86 ± 0.86	5.06 ± 0.93	<0.001 *
Distal	3.94 ± 0.87	5 ± 0.87	<0.001 *
Tendinosis	0	12 (13)	0.008 *
Achilles tendon entheses			
Structural abnormalities	0	13 (14.1)	
Retrocalcaneal bursitis	2 (4.2)	7 (7.6)	0.005 *
Bone erosions	0	11 (12)	0.716
Calcifications			0.016 *
Mild	1 (2.1)	6 (6.5)	
Moderate	1 (2.1)	14 (15.2)	0.015 *
Severe	0	4 (4.3)	

* *p* < 0.05.

**Table 3:** Univariate logistic regression analysis of factors associated with US abnormalities of the Achilles tendon in this study’s HD patients.

Variable	OR	95% CI	*p*
Age	1.062	1.022–1.104	0.002 *
Gender			
Female	Ref	Ref	Ref
Male	2.717	1.131–6.531	0.025 *
Weight	1.000	0.972–1.029	0.979
Height	0.981	0.911–1.056	0.604
Body mass index	1.007	0.932–1.088	0.861
Parathyroid hormone	0.999	0.998–1.000	0.259
Serum calcium	0.729	0.427–1.243	0.246

* *p* < 0.05, OR: odds ratio, CI: confidence interval

## Data Availability

The datasets generated during and/or analyzed during the current study are available from the corresponding author on reasonable request.

## Abbreviations

Ca	calcium
CBC	complete blood count
CRF	chronic renal failure
ESRD	end-stage renal disease
GSUS	grayscale ultrasound
GUESS	Glasgow Ultrasound Enthesitis Scoring System
HD	hemodialysis
HRQoL	health-related quality of life
iPTH	intact parathyroid hormone
MSUS	musculoskeletal ultrasound
PO4	phosphorus
